# Characterization and Biotechnological Potential of Intracellular Polyhydroxybutyrate by *Stigeoclonium* sp. B23 Using Cassava Peel as Carbon Source

**DOI:** 10.3390/polym13050687

**Published:** 2021-02-25

**Authors:** Murilo Moraes Mourão, Luciana Pereira Xavier, Ralph Urbatzka, Lucas Barbosa Figueiroa, Carlos Emmerson Ferreira da Costa, Carmen Gilda Barroso Tavares Dias, Maria Paula Cruz Schneider, Vitor Vasconcelos, Agenor Valadares Santos

**Affiliations:** 1 Laboratory of Biotechnology of Enzymes and Biotransformations, Institute of Biological Sciences, Federal University of Pará, 66075-110 Belém, Pará, Brazil; lpxavier@ufpa.br; 2 Interdisciplinary Centre of Marine and Environmental Research—CIIMAR, University of Porto, 4450-208 Porto, Portugal; rurbatzka@ciimar.up.pt (R.U.); vmvascon@fc.up.pt (V.V.); 3 Laboratory of Oils of the Amazon, Guamá Science and Technology Park, Federal University of Pará, 66075-750 Belém, Pará, Brazil; lucasfigueiroa57@gmail.com (L.B.F.); emmerson@ufpa.br (C.E.F.d.C.); 4 Laboratory of Materials Processing, Institute of Technology, Federal University of Pará, 66075-110 Belém, Pará, Brazil; cgbtd@ufpa.br; 5 Genomics and Systems Biology Center, Federal University of Pará, 66075-110 Belém, Pará, Brazil; mariapaulacruzschneider@gmail.com; 6 Department of Biology, Faculty of Sciences, University of Porto, 4069-007 Porto, Portugal

**Keywords:** Amazonian microalgae, polyhydroxybutyrate, *Stigeoclonium* sp. B23, cassava peel, characterization, zebrafish embryo toxicity

## Abstract

The possibility of utilizing lignocellulosic agro-industrial waste products such as cassava peel hydrolysate (CPH) as carbon sources for polyhydroxybutyrate (PHB) biosynthesis and characterization by Amazonian microalga *Stigeoclonium* sp. B23. was investigated. Cassava peel was hydrolyzed to reducing sugars to obtain increased glucose content with 2.56 ± 0.07 mmol/L. Prior to obtaining PHB, *Stigeoclonium* sp. B23 was grown in BG-11 for characterization and Z8 media for evaluation of PHB nanoparticles’ cytotoxicity in zebrafish embryos. As results, microalga produced the highest amount of dry weight of PHB with 12.16 ± 1.28 (%) in modified Z8 medium, and PHB nanoparticles exerted some toxicity on zebrafish embryos at concentrations of 6.25–100 µg/mL, increased mortality (<35%) and lethality indicators as lack of somite formation (<25%), non-detachment of tail, and lack of heartbeat (both <15%). Characterization of PHB by scanning electron microscopy (SEM), X-ray diffraction (XRD), differential scanning calorimeter (DSC), and thermogravimetry (TGA) analysis revealed the polymer obtained from CPH cultivation to be morphologically, thermally, physically, and biologically acceptable and promising for its use as a biomaterial and confirmed the structure of the polymer as PHB. The findings revealed that microalgal PHB from *Stigeoclonium* sp. B23 was a promising and biologically feasible new option with high commercial value, potential for biomaterial applications, and also suggested the use of cassava peel as an alternative renewable resource of carbon for PHB biosynthesis and the non-use of agro-industrial waste and dumping concerns.

## 1. Introduction

Almost everything on a daily basis involves the use of plastics. Over the years, since the first cellulose thermoplastic, epoxies, polycarbonates, teflons, silicones, polysulfones, nylons, and other plastics, there has been a rapid increase in the quantity, type and quality of plastics. Despite the various advantages of the use of plastics, such as strength, flexibility, etc., environmental pollution and degradation are still major disadvantages in terms of household and industrial waste dumping, the subsequent increase in accumulation in different environments, and the non-treatment of these materials [1,2]. To overcome this problem, the production and characterization of new biodegradable polymers and green-based materials derived from renewable resources has been identified as an alternative to conventional non-degradable and non-degradable plastics by reducing operating costs and increasing opportunities for industrial applications in the eco-friendly circular economy [3,4].

Poly-3-hydroxybutyrate (PHB) is a linear aliphatic polyester of D(−)-3-hydroxybutyric acid and naturally occurring biopolymer, which is produced by several species of bacteria and cyanobacteria in the presence of excess carbon and limited sources of nitrogen/phosphorus and subsequently stored in the inner membrane or in the cytoplasm of cells combined in the form of circular water-insoluble granules as osmotic granules [5,6,7,8]. PHB is the most famous polyhydroxyalkanoate (PHA) homopolymer and, to date, more than 150 forms of PHA have been studied [9]. PHB has a promising industrial interest due to its biodegradable, elastomeric, thermoplastic, biodegradable, biocompatible, and immunological properties [10,11]. In addition, PHB can be used in the manufacture of plastics for quick and routine use, e.g., bags for the management and reduction of domestic and industrial waste, biosensors, tissue engineering scaffolds, drug carriers for various medical treatments used as biomaterials, and others [12,13,14].

Up to now, Archaea, fungi, several gram-negative and positive bacteria, yeasts, photosynthetic cyanobacteria, and transgenic plants are well known and studied groups reported to produce and store different types of PHB under modified and stressful conditions, but there are few reports of the production of polymers by microalgae species [15,16]. However, microalgae have emerged as potential new producers of polyhydroxybutyrate because they are easy and fast to grow, require a few nutritional quantities to survive, perform photosynthesis by absorbing harmful atmospheric gases, in order to release oxygen, and exhibit a high variability of microalgae species potentially used for the production of biodegradable polymers [17,18]. For example, PHB was detected and highly produced in some species of the genus *Chlorella*, such as *Chlorella fusca*, by the addition of pentoses in cultures, varying in the light intensity and photoperiod to stimulate PHB production with 17.4% *w*/*w*, which is the highest biopolymer accumulation through xylose supplementation and 6 h of light [19]; *Chlorella pyrenoidosa* by optimizing growth conditions in Fogg’s medium and Fogg’s broth under a constant light intensity of 80 Lux with 27% PHB content for 14 culture days obtained through UV photospectrometry at 230 nm [20]; *Chlorella* sp. by extraction and characterization of the PHA by solvent extraction method, which proved to be thermally stable below 260 °C [21]; *Chlamydomonas reinhardtii* was co-transformed with two expression vectors containing *phbB* and *phbC* genes from *Ralstonia eutropha* with the amount of PHB levels ranging from 3.3 µg/g (DWt) to 6 µg/g (DWt) [22]; and *Botryococcus braunii* by optimizing certain factors including pH, temperature and substrate for PHB production using response surface methodology, which obtained 247 ± 0.42 mg/L as the maximum yield of PHB from 60% concentration of sewage waste water as a growth medium at pH 7.5 and 40 °C [23]. In a more in-depth and recent study, identification and optimization of PHB production under nitrogen deprivation and supplementation with commercial salts serving as carbon sources were carried out on the first Amazonian oleaginous filamentous green microalga *Stigeoclonium* sp. B23 [24].

The high cost of large-scale production of PHB is mainly caused by the cost of substrates as carbon sources. In this scenario, several raw materials have been used as carbon sources, such as Kenaf (*Hibiscus cannabinus* L.) lignocellulosic biomass for the synthesis and characterization of PHB using *Ralstonia eutropha* [25]; wheat waste biomass hydrolysate was evaluated as a sole carbon source for PHB synthesis by *Ralstonia eutropha* with a maximum of 74% PHA storage and yield of PHB of about 0.441 g/g of monosaccharides production [26]; weed water hyacinth (WH) biomass was also analyzed for PHB biosynthesis by *Ralstonia eutropha* under supplementation with corn steep liquor as a nitrogen source with WH hydrolysates that enhanced PHB synthesis by 73% [27]. In addition, starchy components such as cassava, potatoes or beets, as well as industrial waste from the starch manufacturing process, have become essential raw materials for the PHB production since they are cheap and available locally, particularly in Brazil [28]. Cassava (*Cassava esculenta*) is a root of tropical origin, commonly found in the American and African continents and in several locals in Thailand. Additionally, about 90% of the cassava root is starch in terms of dry weight [29].

The industrial processing of cassava originates four types of waste: husks, fibers, starch residues, and wastewater effluents. Moreover, cassava residues are composed of cellulose, hemicellulose, and lignin, and when they are hydrolyzed with strong acids such as sulfuric acid (H_2_SO_4_), they produce reducing sugars/ monosaccharides, possibly used in numerous optimization applications since they are rich sources of carbon [30]. D-glucose and D-xylose, both decomposed from cellulose and hemicellulose, respectively, are two main reducing sugars in the hydrolysis acid of lignocellulosic materials. In Brazil, for example, the flour industry uses about 80% to process cassava and approximately 10% of the waste eliminated is husks [31]. Cassava peels are rich in organic compounds, which indicates a high potential for the metabolization and synthesis of PHB [29]. However, the cassava peel as an industrial and local waste from several agricultural Brazilian communities has been poorly explored regarding its use as a carbon source for the synthesis of PHB. There are a few studies concerning cassava starch hydrolysate (CSH) as a carbon source, and several species belonging to the genus *Pseudomonas* were explored in this regard when using CSH for synthesis of PHB [32]. D-glucose has been widely used for the cultivation and optimization of biomass production in several microalgae species and *Chlorella* species [33].

The physicochemical and morphological characterization of PHB provide a clear understanding of the polymeric properties when compared to polymers of petrochemical origin and they have not been already well-developed in bacteria biopolymers [10]. Marine cyanobacteria and microalgae have become more sensitive to this low manipulation and offer further research that confirms the large and useful properties of these polymers for later use in medical applications such as drug carrier biomaterials, tissue engineering scaffolds, and others [21,24,34,35,36]. The aim of this article was therefore to study the chemical, physical, morphological, and biological characterization of a novel biotechnological polyhydroxybutyrate by microalgae *Stigeoclonium* sp. B23, using residues of hydrolyzed cassava peel as a carbon source for conversion to PHB. Overall, the research presented focused on providing an appropriate analysis of PHB as a biomaterial nanoparticle molecule in order to recommend it for future medical applications by analyzing its safety with the fish embryo acute toxicity test (FET).

## 2. Materials and Methods

### 2.1. Cassava Peel Pretreatment

The cassava peel used in the present study was collected at the Regional factory, located in Castanhal, Pará, Brazil (1°20′14.8″ S 47°44′55.1″ W). Before pretreatment, the amount of residue from the cassava peel was weighed at a total of 150 g. The first step was to sterilize the waste in an autoclave at 121 °C for 20 min. The material was then dried in an oven at 50 °C for 24 h to remove any traces of liquid. Pretreatment of the residue peel occurred in a laboratory processor under maximum rotation for a period of 5 min in 7 cycles until the maximum amount of powder was obtained. During the treatment of the cassava peel, two forms were obtained: powder and granular. At each cycle, the residue powder was filtered through a 260 μm stainless steel sieve, producing both powder (89.14 g) and non-pulverized granular cassava peel (47.02 g). The cellulose-based powder of the cassava peel had a gray appearance, while the granular powder had a more resistant and brown appearance.

### 2.2. Cassava Peel Hydrolysis

Hydrolysis was used to treat both the powder and the granular cassava peel in order to obtain reducing sugars. The hydrolysis process was based on the methodology of Zhang et al., (2011) [30], which analyzed the best hydrolysis parameters such as temperature, sulfuric acid concentration, and hydrolysis time to obtain maximum sugars, concluding that the best values were 170 °C, 4% (*w*/*w*), and 30 min respectively. These parameters have therefore been used in the present work. To *Stigeoclonium* sp. B23 cultivation, pH was adjusted to 7.0 with 1 N NaOH solution. The cassava peel hydrolysate (CPH) supernatant was used to determine the glucose composition of glucose by the colorimetric method of dinitrosalicylic acid (DNS), which is based on the reduction of 3,5-dinitrosalicylic acid to 3-amino-5-nitrosalicylic acid [37]. A standard curve of glucose was assayed in a range between 0.4 to 4 mmol/L [37]. The values were indicated by means of the three analytical readings.

### 2.3. Media and Growth Conditions

*Stigeoclonium* sp. B23 was provided by Genomics and Systems Biology Center (CGBS) of Federal University of Pará (Belém, Brazil). Initially, microalga was grown in Erlenmeyers flask of 2 L with BG-11 medium [38] for 30 days: 1.5 g/L NaNO_3_, 0.04 g/L K_2_HPO_4_, 0.075 g/L MgSO_4_·H_2_O, 0.036 g/L CaCl_2_·2H_2_O, 0.006 g/L citric acid, 0.006 g/L ferric ammonium citrate, 0.001 g/L Na_2_EDTA, 0.02 g/L sodium carbonate; micronutrients: 2.86 g/L H_3_BO_3_, 1.81 g/L MnCl_2_·4H_2_O, 0.222 g/L ZnSO_4_·7H_2_O, 0.39 g/L Na_2_Mo_4_·2H_2_O, 0.079 g/L CuSO_4_·5H_2_O, and 0.049 g/L Co(NO_3_)_2_·6H_2_O. After this period of adaptation, microalga was cultivated again in 2 L of BG-11 nitrogen-deprived (BG-11_0_) and with different concentrations of sodium nitrate and CPH for PHB production (Table 1). The cultures were again incubated for 30 days at a controlled temperature of 25 °C for a photoperiod of 12 h dark/12 h light, with a pH of 7.

Additionally, *Stigeoclonium* sp. B23 was also grown in nalgene bottles of 20 L of Z8 medium [39]: 46.7 g/L NaNO_3_, 5.9 g/L Ca(NO_3_)_2_·4H_2_O, 2.5 g/L MgSO_4_·7H_2_O, 2.8 g/L FeCl_3_·6H_2_O, 0.1 L HCl (0.1 N), 3.9 g/L EDTA, 0.1 L NaOH (0.1 N), 0.33 g/L Na_2_WO_4_.2H_2_O, 0.88 g/L (NH_4_)_6_Mo_7_O_24_·4H_2_O, 1.2 g/L KBr, 0.83 g/L KJ, 2.87 g/L ZnSO_4_·7H_2_O, 1.55 g/L Cd(NO_3_)_2_·4H_2_O, 1.46 g/L Co(NO_3_)_2_·6H_2_O, 1.25 g/L CuSO_4_·5H_2_O, 1.98 g/L NiSO_4_(NH_4_)_2_SO_4_·6H_2_O, 0.41 g/L Cr(NO_3_)_3_·9H_2_O, 0.089 V_2_O_5_, 9.48 g/L KAl(SO_4_)_2_·12H_2_O, 3.1 g/ L H_3_BO_3_, and 2.23 g/L MnSO_4_·H_2_O under three different concentrations of sodium nitrate (100%: 46.7 g; 25%: 11.675 g and 2.5%: 1.1675 g), at a temperature of 25 °C, with a photon flux in the range of 10–30 µmol/m² s, and both continuous and intermittent aeration for 45 days to obtain increased biomass for polymer production and further analysis of PHB nanoparticles.

### 2.4. Extraction and Quantification of PHB Content

PHB was extracted from 60 mg of freeze-dried biomass by adding 5 mL of sodium hypochlorite to the biomass for 2 h in a water bath. The mixture was homogenized at 5000 rpm for 15 min, and the pellet was washed with 0.5 mL of deionized water and 0.5 mL of acetone and ethyl alcohol solution. The precipitate was dissolved by adding 5 mL of hot chloroform to extract PHB [40]. For Z8 media, the biomass yield (1), PHB content (2), and PHB yield (3) were calculated through the equations below:

(1) $$\mathrm{BMP}\;=\;\frac{M_{\mathrm{BIOMASS}}}{V}$$

(2) $$\mathrm{PHB}\;=\;\frac{M_{\mathrm{PHB}}}{M_{\mathrm{BIOMASS}}}\;\times \;100$$

(3) $$P_{\mathrm{PHB}}\;=\;\mathrm{BMP}\;\times \;\mathrm{PHB}$$

where, M_BIOMASS_ (g): dry biomass; V (L): volume of the culture medium; PHB (%): PHB yield; and M_PHB_ (g): PHB mass.

### 2.5. Statistical Analysis

Microalgae biomass and PHB quantity of means and standard deviations were calculated and performed in triplicates. Two-way variance analysis (ANOVA) was used to test differences in GraphPad Prism 6.01 software (GraphPad Software Inc., San Diego, CA, USA) followed by Tukey’s multiple comparison test to identify statistically significant pairwise differences. The statistical significance level for the assessment of differences between means was *p* = 0.05 overall.

### 2.6. PHB Characterization

#### 2.6.1. Scanning Electron Microscopy (SEM)

The PHB samples were previously freeze-dried to eliminate liquid traces and subsequently metallized with gold for 1.5 min (approx. 15 nm) using Emitech K550X (Montigny-le-Bretonneux, France). The morphology and fractured surfaces of PHB were evaluated using a Zeiss EVO-MA-10 SEM (Oberkochen, Germany) equipment. The operating conditions were: electron beam current = 100 µA, constant acceleration voltage = 1.5 and 5 kV, and working distance = 9 to 10 mm.

#### 2.6.2. Differential Scanning Calorimetry (DSC)

The thermal properties of PHB were determined using the DSC-60 calorimeter (Shimadzu, Kyoto, Japan). Approximately 2.0 mg of extracted PHB was exposed to a temperature profile over 24 °C to 300 °C at a heating rate of 10 °C/min and under nitrogen atmosphere at a flow rate of 50 mL/min. The melting temperature (Tm), glass transition temperature (Tg), and melting enthalpy (∆H_m_) of PHB were calculated by subtracting the baseline in the OriginPro software (OriginLab Corporation, Northampton, MA, USA), which consists of the minimum endotherm peak for T_m_ and the ratio between the area of the endotherm and the PHB mass for ∆H_m_. The degree of crystallization (Xc) (4) was determined using the equation below:

(4) $$X_{c}\;(\%)\;=\;\frac{\Delta H_{m}}{\Delta H_{m}^{0}}\;\times \;100$$

where, $\Delta H_{m}^{0}$ is the melting enthalpy for 100% PHB crystallinity (146.6 J/g), and $\Delta H_{m}$ is the melting enthalpy calculated by the DSC thermograms [41].

#### 2.6.3. Thermogravimetric Analysis (TGA)

The thermal stability of the PHB was performed on the thermal analyzer DTG-60H (Shimadzu, Kyoto, Japan) under a nitrogen atmosphere at a flow rate of 50 mL/min. Based on the weight loss of each sample component, a sample composition was estimated for PHB polymer. Approximately 1.4–2.1 mg of PHB was placed in a platinum pan. Initially, the sample was kept at 25 °C for 5 min and heated to a temperature of 800 °C at a heating flow of 10 °C/min.

#### 2.6.4. X-ray Diffraction Analysis (DRX)

A PANalytical diffractometer (Malvern, UK), Empyrean model, was operated at 40 kV and 35 mA with an X-ray wavelength of 1,789010 Å (Co Kα radiation). Diffraction profiles were recorded by scanning 2θ from 5.0° to 50° with a 0.0263° resolution and a 59.92 s exposure per time. The diffractograms interpretation was performed using the software X’Pert HighScore Plus Version 3.0e (3.0.5) (PANalytical B.V., Almelo, The Netherlands). The crystallite size L (Å) was determined for the highest peaks using the Scherrer Equation (5) below:

(5) $$L\;=\;\frac{K\;\lambda }{\beta \;\cos\theta }$$

where, K is the Scherrer constant (0.94 for spherical crystallites with cubic symmetry); λ is the X-ray wavelength (Co Kα = 1.789010 Å); β is s the FWHM (full width at half maximum) in radians; and θ is the Bragg angle [42].

### 2.7. PHB Nanoparticles Preparation

The PHB nanoparticles treatment was carried out with 1 N of NaOH to break the ester bonds of the polymer. This hydrolysis generates more hydroxyl groups and, consequently, increases the hydrophobicity, improving cell adhesion to the surface of the biomaterial. The PHB nanoparticles were synthesized using a modified nanoprecipitation method proposed by Shakeri et al. 2014 [43]. It consists of dissolving 5 mg of the PHB polymer in 5 mL of trifluoroethanol (TFE), heating, and stirring the solution at 50 °C until the formation of a transparent organic phase. Gradually, this phase was poured into a beaker containing 20 mL of acetone and 1% Tween 80 (*v*/*v*) as a surfactant, using a syringe (22G needle) and under a slight agitation. Subsequently, the solution was centrifuged and the nanoparticle pellet was dried in a Labconco Speedvac (Kansas, MO, USA) overnight.

#### Fish Embryo Acute Toxicity (FET) Test

Fish Embryo Acute Toxicity (FET) Test was conducted in accordance with OECD guideline 236 to analyze the toxicity of PHB nanoparticles on fertilized zebrafish embryos [44]. Twenty embryos per experimental group (one embryo per well) were exposed to five PHB nanoparticles concentrations (6.25, 12.5, 25, 50, and 100 µg/mL) in a 24-well plate for 96 h. 3,4-dichloroaniline (4 mg/L) was used as positive control, and negative (egg water) and solvent control (acetone 0.1%) were included. Four important toxicity parameters were observed every 24 h in the stereomicroscope (Olympus, SZX10, Hamburg, Germany): (a) coagulation of fertilized eggs, (b) lack of somite formation, (c) lack of detachment of the tail-bud from the yolk sac, and (d) lack of heartbeat. At the end of the test, acute toxicity was evaluated using the results obtained in any of the four observed parameters. The approval by an Ethics Committee was not necessary as the chosen procedures were not considered animal experimentation according to the EC Directive 86/609/EEC for animal experiments.

## 3. Results and Discussion

### 3.1. Glucose Composition of CPH during Cultivation

**Figure 1:**
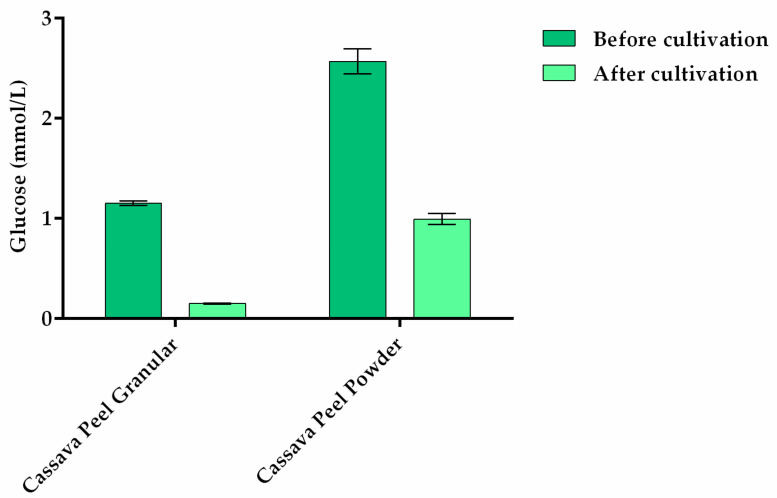
Glucose concentration before and after cultivation of *Stigeoclonium* sp. B23 in BG-11 supplemented with granular and powdered cassava peel. The cultivation of microalga occurred for 30 days. Data are the mean ± SD of three replicates.

As shown in Figure 1, the composition of glucose from cassava peel hydrolysate was determined by the DNS method. It was evaluated which process would provide more glucose to *Stigeoclonium* sp. B23 during cultivation. A specific BG-11 culture was selected whose sodium nitrate concentration was 1.5 g/L (100% NaNO_3_ of BG-11 standard medium) and supplemented with the 1.0 g/L of CPH. One milliliter of BG-11 media before and after cultivation was collected for measurement using a standard glucose curve (Figure S1). The highest concentration of glucose after hydrolysis and before cultivation was obtained with powder cassava peel with 2.56 ± 0.07 mmol/L. Conversely, the maximum glucose concentration of unpulverized granular cassava peel was 1.14 ± 0.01 mmol/L. The glucose amount of powder hydrolysis approximately increased 2-fold more than the non-pulverized granular hydrolysis. However, the consumption of glucose from the hydrolysis of non-pulverized granular was higher with 86.45% and the powdered cassava peel was 61.33%. This can be explained by the fact that acid hydrolysis can improve the porosity of the substrate by using cellulose and hemicellulose, making the substrates more available for enzymes as well as increasing the anaerobic digestion process and subsequent metabolization of sugars by photosynthetic microorganisms such as microalgae and cyanobacteria [45].

Xylose is the second reducing sugar product of acid hydrolysis after glucose synthesis. They are the main products of this reaction. However, in addition to these, other products of acid hydrolysis are generated such as arabinose, acetic acid, and toxic inhibitors, i.e., furans like furfural and 5-hydroxymethylfurfural (HMF) [30]. It is known that inhibitory compounds such as furans can cause adverse effects when formed by the biodegradation of sugars. These inhibitory and toxic effects have been detected in yeasts, bacteria and methane-producing microorganisms [46]. Moreover, the parameters (170 °C reaction temperature, 4% H_2_SO_4_, and 30 min for reaction time) used in the present work increase the concentration of reducing sugars and furans [30]. Nevertheless, these toxic components did not exhibit toxicity and inhibition during the cultivation of *Stigeoclonium* sp. B23, confirmed by glucose consumption by the microalga and subsequent recovery of biomass. These findings demonstrate that *Stigeoclonium* sp. B23 survives in relative extreme environments, but is rich in nutrients such as nitrogen and phosphorus, which are available in BG-11 standard medium [47,48]. Cheap carbon sources, such as agricultural waste, like cassava peel, may decrease additional costs due to pre-treatment steps like acid hydrolysis, shortening the cultivation time and purifying the final product. In addition, carbon sources as raw materials, such as waste from cassava processing, become attractive alternatives to expensive carbon sources [49].

### 3.2. Biomass and PHB Yield

The effects of optimization of both cultivations and PHB production on *Stigeoclonium* species are still poorly described in the literature. In order to improve PHB storage, oleaginous microalgae must accumulate high lipid levels [19]. PHB production occurs when at least one essential nutrient such as nitrogen is in deprivation and enriched with available carbon sources in the culture medium [6]. In addition, as a consequence of this environment, there is a tendency for *Stigeoclonium* sp. B23 not to increase biomass and, consequently, microalga produces PHB efficiently [24]. As shown in Table 2, the biomass yield was the highest in the Z8 standard medium. ANOVA test proved to be significant in all sources of variations of each calculated parameter and Z8 modified media (F = 58.83, *p* < 0.0001) (Table S1), which is explained by the inversely proportional relationship between the amount of nitrogen and PHB production. In Z8 media with 25% and 2.5% of sodium nitrate, 52.28% and 33.98% increased biomass were obtained when compared to standard medium, respectively. The highest concentration of PHB was obtained in Z8 medium with 25% of sodium nitrate (12.16% *w*/*w*) during the lowest level of biomass (0.80 g/L) and followed by Z8 medium with 2.5% of sodium nitrate (8.90% *w*/*w*) by the lowest biomass quantity (0.52 g/L). Increased PHB by both media were 85.71% and 69.56%, respectively. These results were comparable to Cassuriaga et al. 2018, in which 17.4% *w*/*w* PHB concentration was obtained during the lowest level of biomass of *Chlorella fusca* with 0.21 g/L [19].

Oxygenic photosynthesis in microalgae produces ATP and NADPH by transferring electrons in photosystems. The balance between the production of ATP and NADPH plays an important role for the carbon assimilation in the Calvin–Benson–Bassham (CBB) cycle, which provides precursors for carbohydrate biosynthesis [50]. In addition, NADPH is also used in lipid biosynthesis, as well as biotic and abiotic stress affect the functioning of photosystem I by modifying the ability to produce ATP and NADPH [50]. Reducing sugars assimilation by microalgae species requires the presence of both transport mechanisms and high-affinity of D-xylose and D-glucose [51]. In bacteria, this process leads to the growth of NADPH-dependent production by the Tricarboxylic Acid Cycle (TCA), and as a result of this and in response to nitrogen deprivation, PHB synthesis occurs through the conversion of acetoacetyl-CoA into D-3-hydroxybutyrate molecules, which is the precursor of cytoplasmic inclusions of PHB [19,52].

As a result of efficient cultivation with glucose supplementation, it may also be responsible for the increase of NADPH and acetyl-CoA, which are essential substrates for the enzymatic activity of acetoacetyl-CoA reductase, the enzyme responsible for the conversion of acetoacetyl-CoA into D-3-hydroxybutyrate molecules [8]. The excess of NADPH may be responsible for biopolymer synthesis in nitrogen-deprived bacteria cells [52]. In addition, nitrogen and/or phosphorus deprivation can result in decreased protein synthesis, and as a consequence, there is production of ATP, and when this molecule is increased in the intracellular environment, this process can trigger oxidative phosphorylation, causing the growth of precursor coenzymes of polymer production [19,53]. However, this improvement of PHB storage and complete identification of metabolic pathways has still been barely explored in the group of microalgae, and there are no reports on species of the genus *Stigeoclonium* regarding biochemical studies of metabolic pathways.

### 3.3. PHB Nanoparticles Toxicity

**Figure 2:**
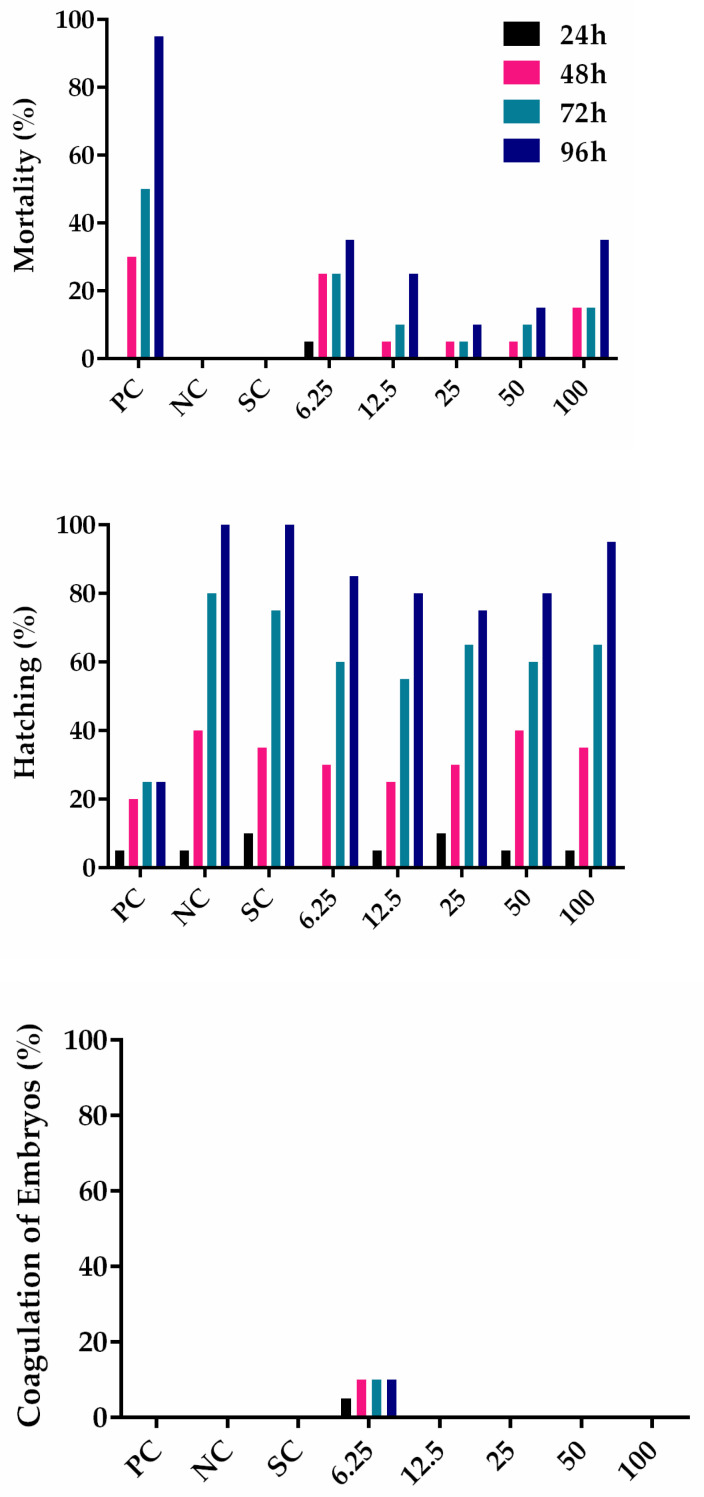

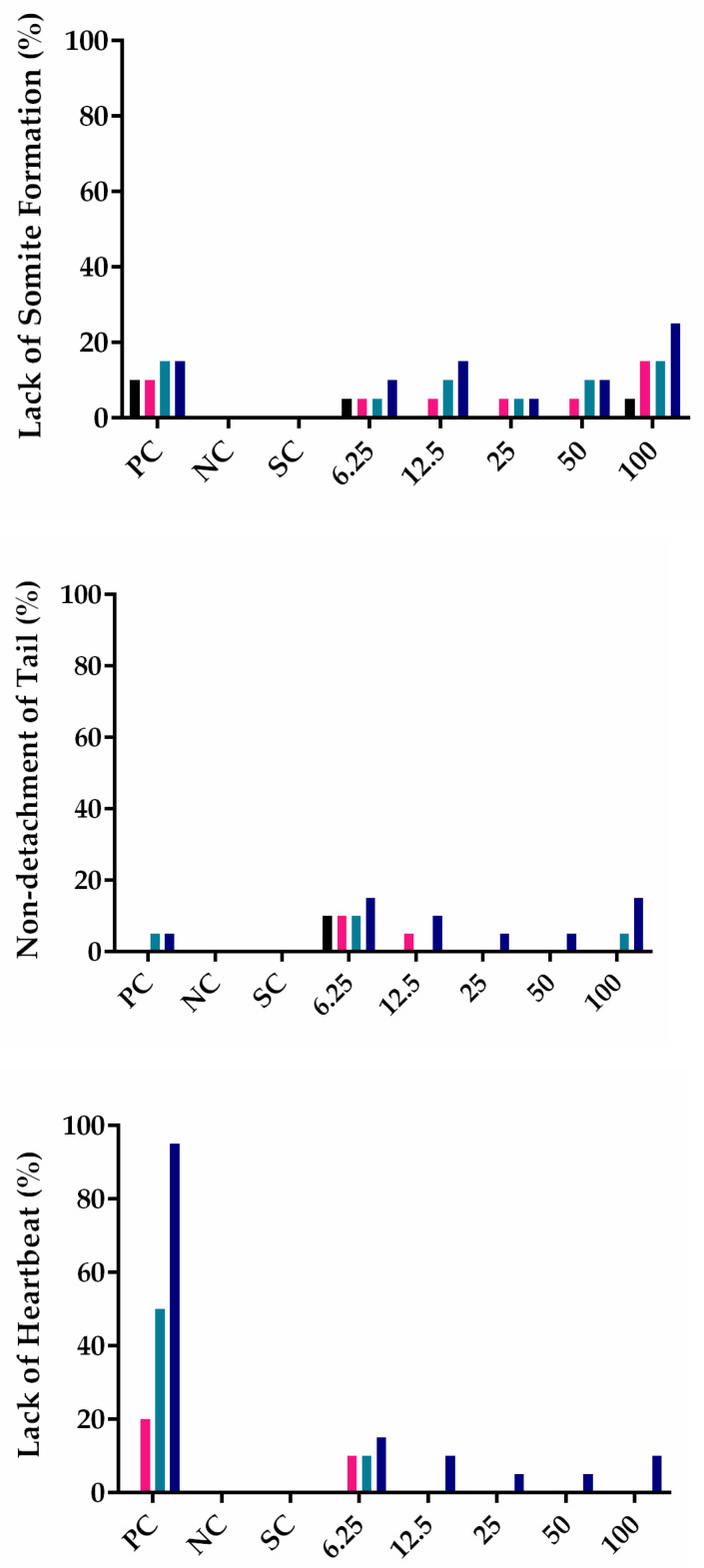
Results from fish embryo acute toxicity test (FET). Cumulative results in percentages are given for mortality, coagulation of embryos, lack of somite formation, hatching, non-detachment of tail, and lack of heartbeat. Data represent 20 embryos per group (1 embryo/well) exposed for 24 h, 48 h, 72 h, and 96 h. PC positive control, NC negative control, SC solvent control, and concentrations of PHB nanoparticles (6.25–100 µg/mL). The experiment was conducted once.

Several types of PHB have great potential in the area of nanotechnology since they have physicochemical properties similar to synthetic polymers and are naturally biodegraded, biocompatible, and being used as drug carriers and scaffolds for tissue engineering due to good mechanical strength, biocompatible nature, nontoxic, and elastomeric properties [54]. In order to analyze the cytotoxicity of PHB, the zebrafish embryo acute toxicity test was applied for the first time to visualize its biological safety by PHB nanoparticles (Figure 2). Mortality was increased between 10 and 35% by PHB nanoparticles at concentrations between 6.25 and 100 µg/mL after 96 h exposure. However, a linear dose-dependency was not observed, and hence a LC_50_ value could not be calculated. It is known that nanoparticle toxicity is complex, depending not only on the concentration, but on size, shape, formation of aggregates, and surface charge variations [55]. Hatching rate increased normally during the exposure time for the solvent and negative controls and was slightly decreased by 20% in PHB nanoparticle exposure groups. The PHB nanoparticles induced a lack of somite formation between 5 and 25% of zebrafish embryos after 96 h exposure, at a similar effect strength as the positive control 3,4-dichloroaniline. Five to 15% of zebrafish embryos did not show detachment of their tails and lacked a visible heartbeat. No differences in toxicity parameters were observed for PHB nanoparticles between the chorion-protected life stages before 48 h of development and hatched embryos until 96 h. Such a protective role of the chorion and toxicity differences is often observed for nanoparticles, e.g., described for SiO_2_ nanoparticles [56]. In contrast to the observed toxicity on zebrafish embryos, PHB is described as a non-toxic biopolymer [57], and PHB films such as poly(3-hydroxyoctanoate) (PHO) were not toxic on L929 cells and not pro-inflammatory [58]. However, PHB biopolymers decreased oxidative stress markers in the blue mussel [59].

### 3.4. Characterization Analysis

#### 3.4.1. Morphology of Microalgal PHB

**Figure 3:**
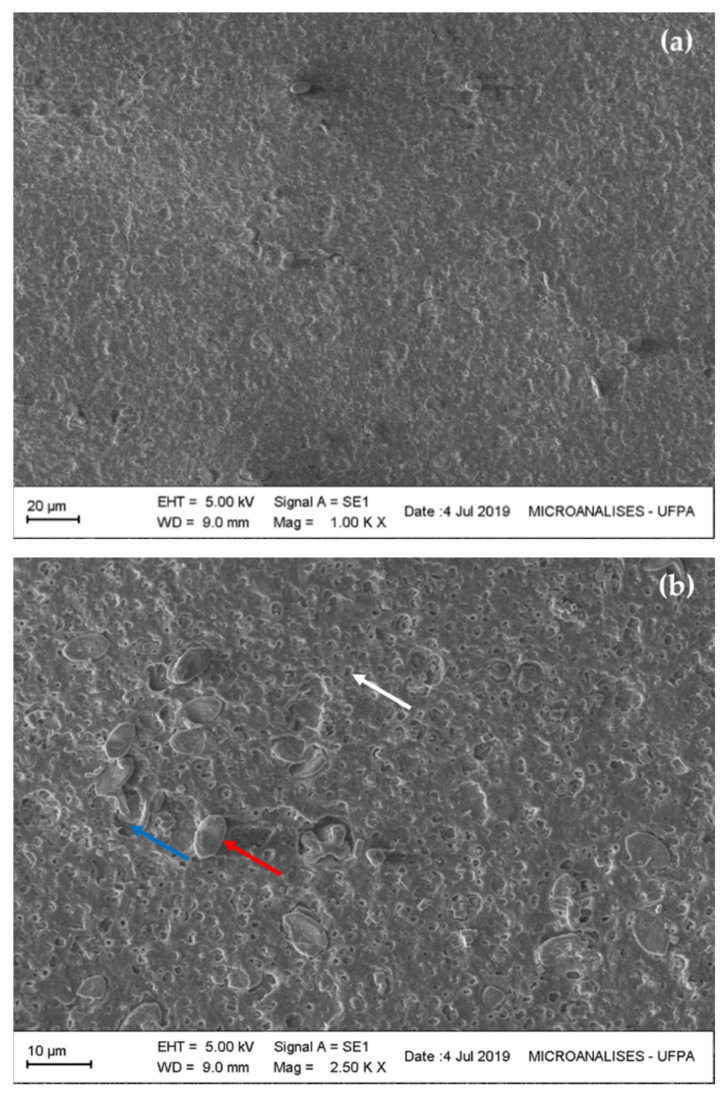

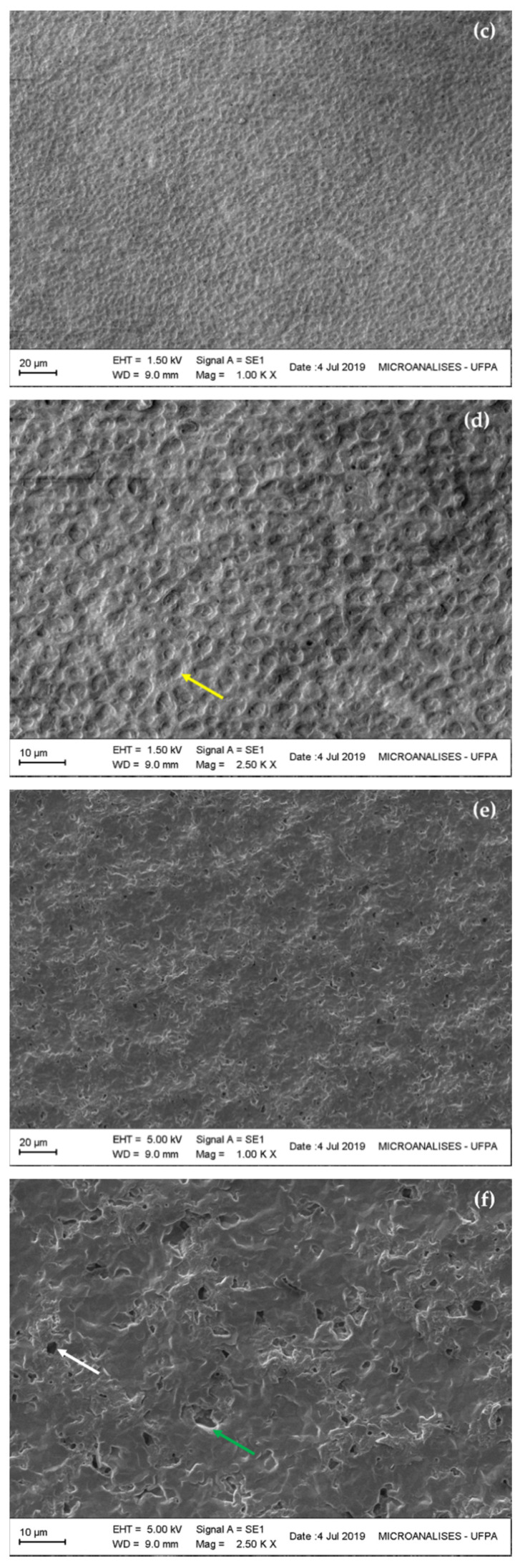

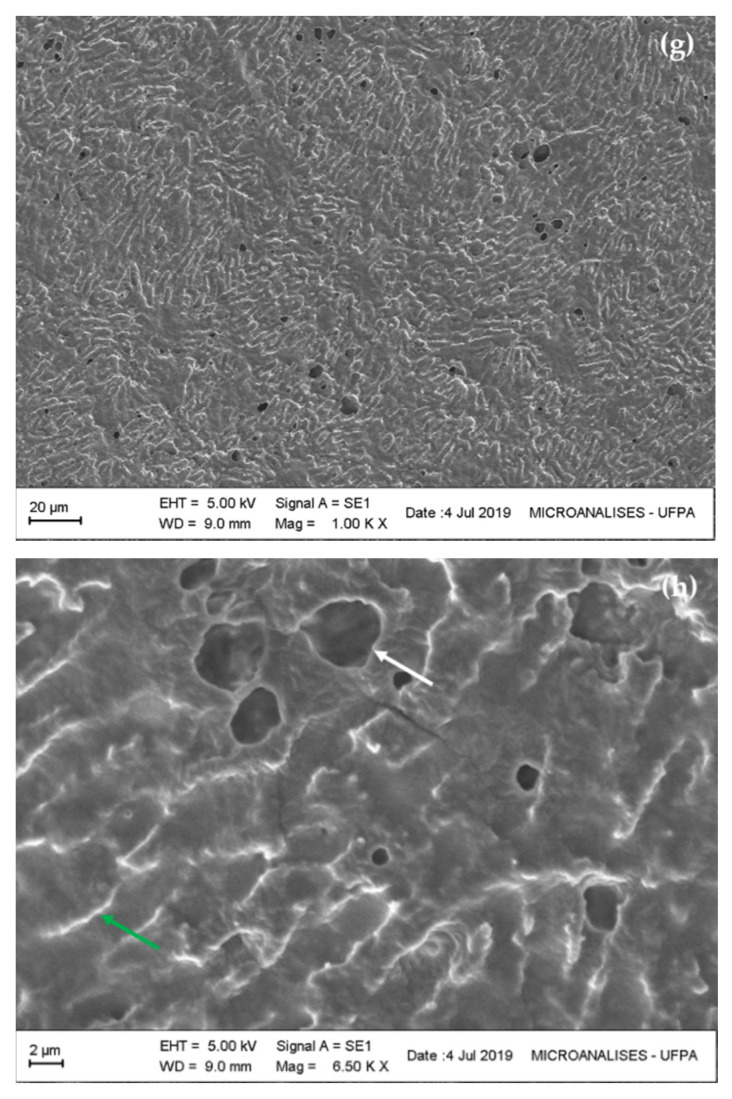
SEM images of the fractured and porous surface of PHB extracted from oleaginous microalga *Stigeoclonium* sp. B23. (**a**,**b**): BG-11 without sodium nitrate; (**c**,**d**): cultivation 1; (**e**,**f**): cultivation 2; (**g**,**h**): cultivation 3. Blue arrow: empty grain holes; red arrow: non-attached grains; white arrow: small pores; yellow arrow: homogeneous and filled holes; green arrow: agglomerated structures compartment.

Figure 3 shows SEM images of characteristic regions of fractured surface and morphologies of PHB by *Stigeoclonium* sp. B23. PHB was extracted by modifying the microalga cultivation. The PHB surface is naturally brittle, hard, rough, and highly porous due to its crystallinity structure and more ductile fracture behavior [60,61]. Extracted PHB from BG-11 with no sodium nitrate showed a non-rigid, scaly, soft, and easily breakable surface, with non-attached grains and the formation of empty and brittle small pores with different sizes, possibly caused by the non-addition of carbon that could reinforce the polymer, indicating a small open-pore matrix (Figure 3a,b). This development is possibly linked to the initial process of biodegradation, which decreases the quality of PHB, but it improves the flow and metabolization of the polymer [49]. Therefore, degradation of the polymer in the human body through surface erosion makes PHB more suitable and, consequently, can be employed in several applications such as nerve repair implants and as support material for bone inserts [14].

In modified cultivations with sodium nitrate and CPH (Table 1), the PHB extracted exhibited high porosity, spongy surface, less brittle, and more rigid and robust than the polymer previously evaluated. The best PHB surface was observed in the polymer extraction of cultivation 1 (Figure 3c,d), with a porous scaffold pre-assembled and flat surface throughout the sample. The average pore size was not calculated. The irregular presence of fibrils around the pores was also observed, with a non-homogeneous fibrillar appearance in the polymer of cultivation 2 (Figure 3e,f) and homogeneous in the polymer of cultivation 3 (Figure 3g,h). The SEM images clearly show the shift from a brittle-matrix form of morphology to a surface with interpenetrating structures. Therefore, enrichment with excessive carbon sources reinforces the surface of the PHB polymer matrix.

These characteristics of fractured PHB are related to a possible improvement of the polymer surface matrix and its use in the application of cell adhesion for 3D engineering, drug release and cell proliferation studies [14,62]. However, to make PHB polymers biologically active and a suitable biodegradable biomaterials, it is necessary to make them softer and more elastic. In this regard, PHB composites with hydroxyapatite (HA), clay, and copolymers such as poly(3-hydroxybutyrate-co-3-hydroxyvalerate) (PHBV) provide good physical and mechanical properties and increase hydrophobicity and degradation [62]. The PHB improvement from *Stigeoclonium* sp. B23 cultivation with an excessive and cheap carbon source indicates a greater development of the polymer and increases the physical and biological qualities of it, enabling its use as a potential biomaterial.

#### 3.4.2. Thermal and Degradation Behaviour of Microalgal PHB

**Figure 4:**
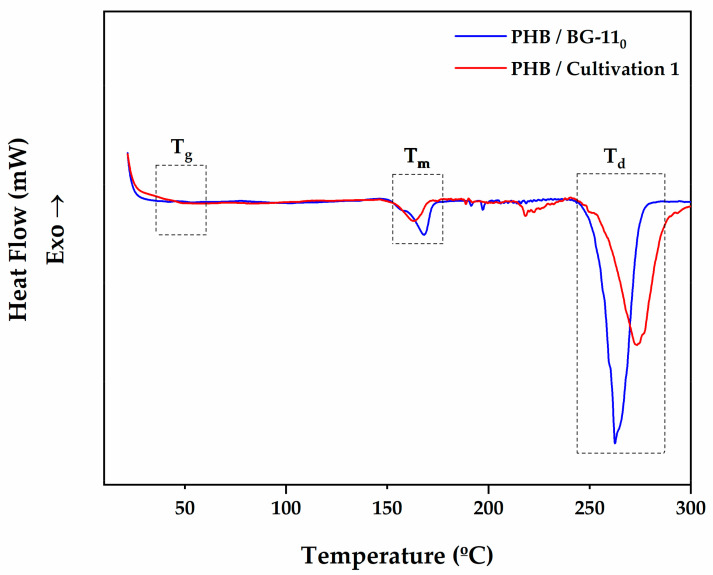
DSC thermograms of PHB obtained from *Stigeoclonium* sp. B23 grown in BG-11 under nitrogen deprivation (BG-11_0_) and modified medium with 0.5 g/L of sodium nitrate and 10 g/L of CPH (Cultivation 1) at a heating rate of 10 °C/min. Tg: glass transition temperature, Tm: melting temperature, Td: degradation temperature. The experiment was conducted once.

DSC analysis of PHB extracted from *Stigeoclonium* sp. B23 was carried out in order to have an understanding of its thermal properties. Table 3 summarizes the thermal properties obtained from the DSC thermograms. As shown in Figure 4, the CPH biopolymer of both cultivations showed two endothermal peaks between 150 and 300 °C. The endothermic peak at the lower temperature is attributed to the melting behavior of the crystalline PHB film. The other endothermic peak at a high temperature is shown, which is probably due to the degradation of the biopolymer. PHB from BG-11_0_ exhibited a melting endotherm at Tm = 168.21 °C and cultivation 1 displayed a melting endotherm at Tm = 164.09 °C. Since the pure PHB has a melting temperature between 170–180 °C, the melting temperatures of the PHB extracted from the microalga biomass were slightly below and within the range between 160–170 °C [63]. However, according to Salgaonkar and Bragança, 2017 [42], there is a possibility of multiple endothermic peaks appearing below the temperature range of the melting temperature for pure PHB. These relatively low temperatures during heating scan occur due to the variation of monomer units of 3HB (3-hydroxybutyrate) and 3HV (3-hydroxyvalerate) forming the copolymer (P(3HB–3HV) [64]. The degradation temperature (Td) peaks for the biopolymer from BG-11_0_ and Cultivation 1 were at 262.54 °C and 274.58 °C obtained by DSC thermograms, and the glass transition temperature (Tg) peaks were at 44.32 °C and 46.04 °C, respectively. Thermal degradation of PHB occurs quickly at temperatures of 190 °C and higher as shown in the present work [65]. Usually, the glass transition temperature occurs between 5–9° [11]. The glass transition temperature values obtained of the extracted PHB from *Stigeoclonium* sp. B23 were higher than the pure PHB and several studies [11,35,66,67]. In addition, a glass transition temperature similar to the present study was found in a strain of *Synechocystis* sp. [34]. Nevertheless, the glass transition temperature of PHB extracted from Lysinibacillus sphaericus was 140 °C, proving that high glass transition temperatures of different types of PHB can be determined in different microorganisms [66]. The melting enthalpy determined during the heating scan was used to calculate the crystallinity rate (%) of the PHB through Equation (4). The melting enthalpies of extracted PHB from BG-11_0_ and Cultivation 1 were 19.74 J/g and 11.03 J/g, respectively. Crystallinity degree (Xc) was also calculated, and it was assumed the melting enthalpy of 100% crystalline PHB to be 146.6 J/g [41]. The crystallinity degree obtained by PHB from *Stigeoclonium* sp. B23 was about 7.52–13.46% lower compared to those detected for other types of PHB [35,66,67,68,69,70]. However, impurities from the extraction process or even the presence of other HB or HV monomers can decrease the crystallinity of the polymer [71]. Moreover, PHBs that have a degree of crystallinity between 60–80% are considered too rigid. The lower the degree of crystallinity of PHB, the greater the number of biotechnological applications in which it can be used, by improving its mechanical and physicochemical properties [67].

**Figure 5:**
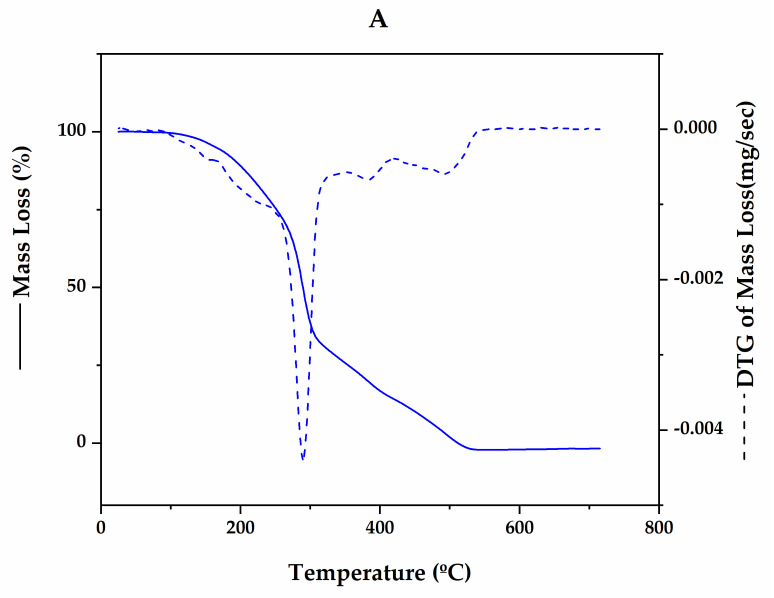

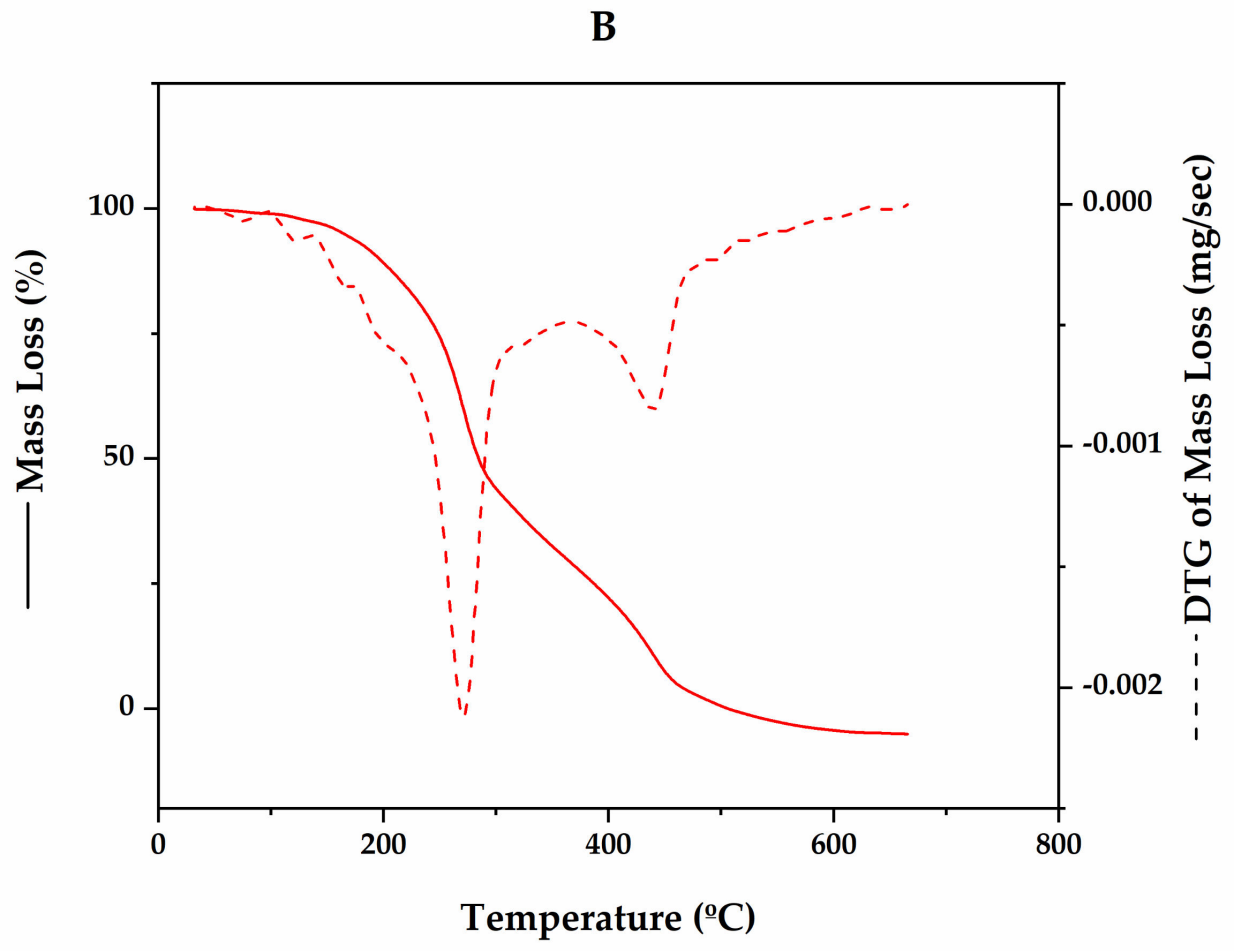
Thermo gravimetric analysis curves at a heating flow of 10 °C/min of PHB produced by *Stigeoclonium* sp. B23 cultivated in (**A**): BG-11 under nitrogen deprivation (BG-11_0_) and (**B**): BG-11 under nitrogen deprivation and supplemented with cassava peel hydrolysate (Cultivation 1). Figures show more than one thermal event. The experiment was conducted once.

Figure 5 shows the associated thermogravimetric and derivative thermogravimetric curves of PHB samples. TGA and dTGA curves showed that the two PHB samples tested underwent more than one thermal event due to the presence of impurities and additives during the polymer extraction process. Thermal degradation of PHB is determined by a random chain break reaction of PHB ester groups to form shorter chains with carboxylic and olefinic terminal groups [35]. In the PHB extracted from the microalga grown in BG-11_0_ (Figure 5A), the initial decomposition temperature (T_onset_) of PHB was 252.90 °C and 225.58 °C in the medium enriched with CPH (Figure 5B). This result shows that the PHB obtained can be thermally stable at temperatures below 220 °C (Table 3). Additionally, thermal degradation was determined during weight loss, in which the losses were 1.467 mg (71.56%) for BG-11_0_ and 1.038 mg (69.99%) for the culture enriched with CPH. These data indicate the presence of impurities or additives remaining from the PHB extraction process [72]. The degradation temperature (Td) peaks for the biopolymer from BG-11_0_ and Cultivation 1 were at 271.72 °C and 254.20 °C obtained by TGA and dTGA curves, which are relatively similarly obtained from DSC curves (Figure 4). The thermal degradation around 300 °C is mostly related with the ester cleavage of PHB by β-elimination reaction [70]. The presence of two new thermal degradation events was also observed due to the presence of impurities and additives previously cited in BG-11_0_ with T_onset_ at 377.15 °C and 454.03 °C and one new thermal degradation event in PHB extracted CPH cultivation with T_onset_ at 413.58 °C. The thermal degradation results of the microalgal PHB are similar to the thermal properties of the commercial PHB obtained by Lee et al. 2002, [73]. To make the PHB matrix thermally more resistant, it is necessary to reinforce it with subunits of 3HV and 4HB, resulting in a material with good properties and potentially can be applied in a range of biotechnological applications [74]. Thus, the results presented suggest that the PHB obtained from the Amazonian microalga *Stigeoclonium* sp. B23 produces a higher percentage of pure biopolymer of PHB and can potentially be used in several areas of current biotechnology, such as in the manufacture of biodegradable biomaterials.

#### 3.4.3. Cristallinity of Microalgal PHB

**Figure 6:**
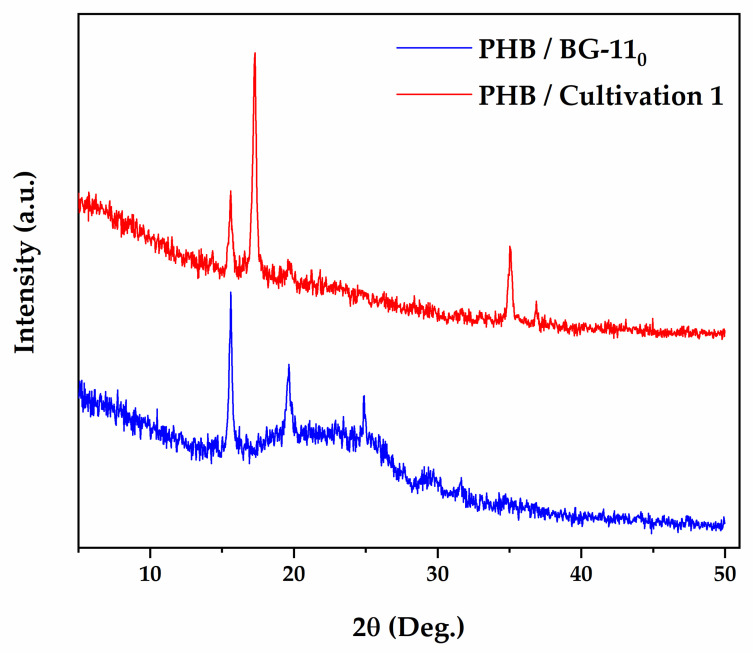
Comparison of X-ray diffractograms of PHB polymer extracted from *Stigeoclonium* sp. B23 cultivated in BG-11 under nitrogen deprivation (BG-11_0_) and complemented with cassava peel hydrolysate (Cultivation 1). Microalgal PHB biopolymer showed characteristic reflection peaks at 2θ. The experiment was conducted once.

Figure 6 shows the XRD patterns of the PHB samples from *Stigeoclonium* sp. B23. The diffractograms showed that these biopolymers were naturally semicrystalline and the presence of only one crystalline phase was also observed. The diffraction profile of microalgal PHB samples is analogous to Poly[(R,S)-β-hydroxybutyrate] obtained through the HighScore Plus software database. Microalgal PHB biopolymer showed characteristic reflection peaks at 2θ = 15.624°, 19.622°, 26.04°, and 31.67° for the polymer from BG-110 and 15.59°, 17.28°, 19.62°, and 35.05° from Cultivation 1. These values are similar to that obtained with different types of PHB [42,66,70,75,76]. The crystallite domain size was calculated using the Scherrer Equation (5). Non-wide and long peaks were observed, suggesting highly-oriented crystallites of large size: 2337 Å and 2473 Å for the total sum of crystallites, respectively. However, there was a change in peak positions and a decrease in peak intensity in CPH polymer when compared with PHB from nitrogen deprivation. For example, this variation in the size of the peaks was observed around the peak 15°, with increased intensity in BG-110 culture and whose crystallite size was 991 Å, which decreased considerably to 473 Å of the CPH polymer. The same occurs with the peak around degree 19, with 378 Å and 154 Å, respectively. Peak shifts were also observed. The intensity of the 19° peak was higher in the PHB extracted from nitrogen-deprived cultivation. Furthermore, this can be explained by the overlap and greater intensity of the 17° peak in the CPH polymer, which obtained 546 Å of crystallite size and was not seen in the PHB from BG-110 culture. This result clearly indicates an inversely proportional relationship between the size of the crystallite and the peak width [42]. Broader peaks showed in the diffractograms do not belong to PHB and are due to the impurities remaining during sample preparation and subsequent extraction with solvents.

According to Anbukarasu et al. 2017, the decrease in sample thickness is related to the loss of some peaks that correspond to the orthorhombic crystal planes of PHB, i.e., the (110) and (111), which are linked to the edge-on crystal planes [75]. Thinner PHB films display more anisotropy, in which their physical properties vary with direction and where the crystals are required to increase more specially in the plane of the polymer film direction [77]. This result was obtained in the PHB extracted from the cultivation without sodium nitrate, since its surface was thinner when compared to the PHB extracted from the cultivation with CPH and confirmed by SEM images.

Brazil is one of the main exporting countries of starch and cassava flour and the amount of waste resulting from this processing is high and not properly used. Our results, therefore, suggest and indicate the use of these agro-industrial residues such as cassava peel as a carbon source for bioconversion into PHB in microalgae species. *Stigeoclonium* sp. B23 was able to absorb reducing sugars efficiently and subsequently produced intracellular PHB. The tests and results obtained here were also shown to be similar to the PHB of other species and confirm the potential for the use of biopolymer in several biotechnological applications and mainly in medical applications, since PHB has favorable mechanical properties for use in these areas once its toxicity is low in zebrafish embryos. Currently, studies are focused on the use of several species of bacteria for the bioconversion of lignocellulosic products into biopolymers such as PHB. Thus, the culture optimization of oleaginous green microalgae species using other sources of lignocellulosic raw materials may become essential for the discovery of new types of polyhydroxybuturates. However, the high price of PHB is still a challenge, and this can be mitigated by the use of cheaper substrates and the search for new and more efficient microorganisms that produce biodegradable polymers with similar mechanical and thermal properties or better than petrochemical plastics.

## 4. Conclusions

In the present work, crude cassava peel hydrolysate has been used used as a carbon source for PHB production by Amazonian oleaginous green microalga *Stigeoclonium* sp. B23. The reducing sugar resulting from the hydrolysis of the cassava peel was efficient and productive for the cultivation of microalga in BG-11 medium and gave an increased glucose content by the pretreatment through the acid hydrolysis. Increased polymer production was also observed in Z8 medium with 25% sodium nitrate reduction, obtaining 12.16 ± 1.28% (DWt) of the polymer. First characterization of PHB polymer safety revealed that mortality and some lethality indicators in zebrafish embryos were induced. The physicochemical, biological, thermal, and morphological properties of the PHB extracted from the microalga were similar to the properties of several types of PHB and its copolymers from species of cyanobacteria and bacteria. With these characterization data, PHB obtained from *Stigeoclonium* sp. B23 can be efficiently developed into a biomaterial application, serving as a drug carrier, and this confirmed that this polymer has promising polymeric properties. Both improved cultivation and physicochemical parameters must be essential in evaluating the growth of microalgae species regarding PHB production.

## Figures and Tables

**Table 1:** Three conditions of culture of *Stigeoclonium* sp. B23 in BG-11 medium with different concentrations of cassava peel hydrolysate (CPH) and sodium nitrate (NaNO_3_).

Cultivation	CPH (g/L)	NaNO_3_ (g/L)
1	10	0.5
2	5.0	1.0
3	1.0	1.5

**Table 2:** Content and yield of biomass and PHB obtained by *Stigeoclonium* sp. B23 cultivation in Z8 culture medium under different concentrations of sodium nitrate. **BMP:** Biomass yield; **PHB:** PHB content; **P_PHB_:** PHB yield. Data are the mean ± SD of three replicates by two-way ANOVA with Tukey Multiple Comparisons Test.

Medium	NaNO_3_ (g)	BMP (g/L)	PHB (%)	P_PHB_ (g/L)
Z8/100%NaNO_3_	46.7	1.53 ± 0.09	0.92 ± 0.01	0.014 ± 0.001
Z8/25%NaNO_3_	11.675	0.80 ± 0.06	12.16 ± 1.28	0.098 ± 0.005
Z8/2.5%NaNO_3_	1.1675	0.52 ± 0.06	8.90 ± 1.96	0.046 ± 0.006

**Table 3:** Thermal and degradation properties of PHB produced by *Stigeoclonium* sp. B23.

	DSC Characterization							
PHB	T*g* (°C)	T*m* (°C)	$\Delta H_{m}$ (J/g)	X*_c_* (%)	T*d* (°C)	T*_onset_* (°C)	T*_endset_* (°C)	$\Delta H_{d}$ (J/g)
BG-11_0_	44.32	168.31	19.74	13.46	271.72	252.90	302.08	186.70
Cultivation 1	46.04	164.09	11.03	7.52	254.20	225.58	289.19	142.66

## Data Availability

The data presented in this study are available on request from the corresponding author.

## Supplementary Materials

The following are available online at https://www.mdpi.com/2073-4360/13/5/687/s1, Figure S1: Standard curve of commercial glucose. UV absorbance 540 nm is plotted as a function of the amount of glucose in mmol/L. Data are the mean ± SD of three replicates, Table S1: ANOVA Table of Calculated Parameter (BMP, PHB and PPHB) and Modified Z8 Media of *Stigeoclonium* sp. B23 at the 95% Confidence Level, Table S2: Tukey’s Post Hoc Multiple Comparisons of Calculated Parameters (BMP, PHB, and PPHB) and Modified Z8 Media of *Stigeoclonium* sp. B23. The Mean Difference is Significant at the 0.05 level.

## References

[1] Chen G.-Q., Patel M.K. (2011). Plastics derived from biological sources: Present and future: A technical and environmental review. Chem. Rev..

[2] Chae Y., An Y.-J. (2018). Current research trends on plastic pollution and ecological impacts on the soil ecosystem: A review. Environ. Pollut..

[3] Yates M.R., Barlow C.Y. (2013). Life cycle assessments of biodegradable, commercial biopolymers—A critical review. Resour. Conserv. Recycl..

[4] Gradíssimo D.G., Xavier L.P., Santos A.V. (2020). Cyanobacterial polyhydroxyalkanoates: A sustainable alternative in circular economy. Molecules.

[5] Jendrossek D., Pfeiffer D. (2014). New insights in the formation of polyhydroxyalkanoate granules (carbonosomes) and novel functions of poly(3-hydroxybutyrate). Environ. Microbiol..

[6] Kushwah B.S., Singh V. (2016). Towards understanding polyhydroxyalkanoates and their use. J. Polym. Res..

[7] Reddy C.S.K., Ghai R., Kalia V. (2003). Polyhydroxyalkanoates: An overview. Bioresour. Technol..

[8] Lee I., Kim M.K., Chang H.N., Park Y.H. (1995). Regulation of poly-β-hydroxybutyrate biosynthesis by nicotinamide nucleotide in *Alcaligenes eutrophus*. FEMS Microbiol. Lett..

[9] Mitra R., Xu T., Xiang H., Han J. (2020). Current developments on polyhydroxyalkanoates synthesis by using halophiles as a promising cell factory. Microb. Cell Factories.

[10] Mostafa Y.S., Alrumman S.A., Otaif K.A., Alamri S.A., Mostafa M.S., Sahlabji T. (2020). Production and characterization of bioplastic by polyhydroxybutyrate accumulating erythrobacter aquimaris isolated from mangrove rhizosphere. Molecules.

[11] Możejko-Ciesielska J., Kiewisz R. (2016). Bacterial polyhydroxyalkanoates: Still fabulous?. Microbiol. Res..

[12] Raza Z.A., Khalil S., Abid S. (2020). Recent progress in development and chemical modification of poly(hydroxybutyrate)-based blends for potential medical applications. Int. J. Biol. Macromol..

[13] Bugnicourt E., Cinelli P., Lazzeri A., Alvarez V. (2014). Polyhydroxyalkanoate (PHA): Review of synthesis, characteristics, processing and potential applications in packaging. Express Polym. Lett..

[14] Gadgil B.S.T., Killi N., Rathna G.V.N. (2017). Polyhydroxyalkanoates as biomaterials. MedChemComm.

[15] Sharma V., Sehgal R., Gupta R. (2021). Polyhydroxyalkanoate (PHA): Properties and modifications. Polymer.

[16] Dobrogojski J., Spychalski M., Luciński R., Borek S. (2018). Transgenic plants as a source of polyhydroxyalkanoates. Acta Physiol. Plant..

[17] Abdo S.M., Ali G.H. (2019). Analysis of polyhydroxybutrate and bioplastic production from microalgae. Bull. Natl. Res. Cent..

[18] Costa S.S., Miranda A.L., de Morais M.G., Costa J.A.V., Druzian J.I. (2019). Microalgae as source of polyhydroxyalkanoates (PHAs)—A review. Int. J. Biol. Macromol..

[19] Cassuriaga A., Freitas B., Morais M., Costa J. (2018). Innovative polyhydroxybutyrate production by Chlorella fusca grown with pentoses. Bioresour. Technol..

[20] Das S.K., Sathish A., Stanley J. (2018). Production of biofuel and bioplastic from chlorella pyrenoidosa. Mater. Today Proc..

[21] Roja K., Sudhakar D.R., Anto S., Mathimani T. (2019). Extraction and characterization of polyhydroxyalkanoates from marine green alga and cyanobacteria. Biocatal. Agric. Biotechnol..

[22] Chaogang W., Zhangli H., Anping L., Baohui J. (2010). Biosynthesis of Poly-3-Hydroxybutyrate (PHB) in the transgenic green alga *Chlamydomonas reinhardtii*. J. Phycol..

[23] Kavitha G., Kurinjimalar C., Sivakumar K., Kaarthik M., Aravind R., Palani P., Rengasamy R. (2016). Optimization of polyhydroxybutyrate production utilizing waste water as nutrient source by *Botryococcus braunii* Kütz using response surface methodology. Int. J. Biol. Macromol..

[24] Mourão M.M., Gradíssimo D.G., Santos A.V., Schneider M.P.C., Faustino S.M.M., Vasconcelos V., Xavier L.P. (2020). Optimization of polyhydroxybutyrate production by amazonian microalga *Stigeoclonium* sp. B23. Biomolecules.

[25] Saratale R.G., Saratale G.D., Cho S.K., Kim D.S., Ghodake G.S., Kadam A., Kumar G., Bharagava R.N., Banu R., Shin H.S. (2019). Pretreatment of kenaf (*Hibiscus cannabinus* L.) biomass feedstock for polyhydroxybutyrate (PHB) production and characterization. Bioresour. Technol..

[26] Saratale G.D., Saratale R.G., Varjani S., Cho S.-K., Ghodake G.S., Kadam A., Mulla S.I., Bharagava R.N., Kim D.-S., Shin H.S. (2020). Development of ultrasound aided chemical pretreatment methods to enrich saccharification of wheat waste biomass for polyhydroxybutyrate production and its characterization. Ind. Crop. Prod..

[27] Saratale R.G., Cho S.-K., Ghodake G.S., Shin H.-S., Saratale G.D., Park Y., Lee H.-S., Bharagava R.N., Kim D.-S. (2020). Utilization of noxious weed water hyacinth biomass as a potential feedstock for biopolymers production: A novel approach. Polymers.

[28] Anjum A., Zuber M., Zia K.M., Noreen A., Anjum M.N., Tabasum S. (2016). Microbial production of polyhydroxyalkanoates (PHAs) and its copolymers: A review of recent advancements. Int. J. Biol. Macromol..

[29] Zhang M., Xie L., Yin Z., Khanal S.K., Zhou Q. (2016). Biorefinery approach for cassava-based industrial wastes: Current status and opportunities. Bioresour. Technol..

[30] Zhang Q., Tang L., Zhang J., Mao Z., Jiang L. (2011). Optimization of thermal-dilute sulfuric acid pretreatment for enhancement of methane production from cassava residues. Bioresour. Technol..

[31] Zeoula L.M., Neto S.F.C., Branco A.F., Prado I.N.D., Dalponte A.O., Kassies M., Fregadolli F.L. (2002). Mandioca e resíduos das farinheiras na alimentação de ruminantes: pH, Concentração de N-NH3 e eficiência microbiana. Rev. Bras. Zootec..

[32] Poomipuk N., Reungsang A., Plangklang P. (2014). Poly-β-hydroxyalkanoates production from cassava starch hydrolysate by *Cupriavidus* sp. KKU38. Int. J. Biol. Macromol..

[33] Bumbak F., Cook S., Zachleder V., Hauser S., Kovar K. (2011). Best practices in heterotrophic high-cell-density microalgal processes: Achievements, potential and possible limitations. Appl. Microbiol. Biotechnol..

[34] Lackner M., Kamravamanesh D., Krampl M., Itzinger R., Paulik C., Chodak I., Herwig C. (2019). Characterization of photosynthetically synthesized poly(3-hydroxybutyrate) using a randomly mutated strain of *Synechocystis* sp. PCC 6714. Int. J. Biobased Plast..

[35] Ansari S., Fatma T. (2016). Cyanobacterial Polyhydroxybutyrate (PHB): Screening, optimization and characterization. PLoS ONE.

[36] Hong K., Beld J., Davis T.D., Burkart M.D., Palenik B. (2020). Screening and characterization of polyhydroxyalkanoate granules, and phylogenetic analysis of polyhydroxyalkanoate synthase gene *PhaC* in cyanobacteria. J. Phycol..

[37] Miller G.L. (1959). Use of dinitrosalicylic acid reagent for determination of reducing sugar. Anal. Chem..

[38] Allen M.M. (1968). Simple conditions for growth of unicellular blue-green algae on plates. J. Phycol..

[39] Kotai J. (1972). Instructions for preparation of modified nutrient solution Z8 for algae. Nor. Inst. Water Res..

[40] Getachew A., Woldesenbet F. (2016). Production of biodegradable plastic by polyhydroxybutyrate (PHB) accumulating bacteria using low cost agricultural waste material. BMC Res. Notes.

[41] Kuntzler S.G., De Almeida A.C.A., Costa J.A.V., De Morais M.G. (2018). Polyhydroxybutyrate and phenolic compounds microalgae electrospun nanofibers: A novel nanomaterial with antibacterial activity. Int. J. Biol. Macromol..

[42] Salgaonkar B.B., Bragança J.M. (2017). Utilization of sugarcane bagasse by halogeometricum borinquense strain E3 for biosynthesis of poly(3-hydroxybutyrate-co-3-hydroxyvalerate). Bioengineering.

[43] Shakeri F., Shakeri S., Hojjatoleslami M. (2014). Preparation and characterization of carvacrol loaded polyhydroxybutyrate nanoparticles by nanoprecipitation and dialysis methods. J. Food Sci..

[44] Organisation for Economic Co-operation and Development (2013). Test No. 236: Fish Embryo Acute Toxicity (FET) Test. OECD Guidel. Test. Chem. OECD Publ..

[45] Kim J., Park C., Kim T.-H., Lee M., Kim S., Kim S.-W., Lee J. (2003). Effects of various pretreatments for enhanced anaerobic digestion with waste activated sludge. J. Biosci. Bioeng..

[46] Palmqvist E., Hahn-Hägerdal B. (2000). Fermentation of lignocellulosic hydrolysates. II: Inhibitors and mechanisms of inhibition. Bioresour. Technol..

[47] Luttenton M.R., Lowe R.L. (2006). Response of a lentic periphyton community to nutrient enrichment at low n:p ratios. J. Phycol..

[48] Marks J.C., Lowe R.L. (1989). The independent and interactive effects of snail grazing and nutrient enrichment on structuring periphyton communities. Hydrobiologia.

[49] Chanprateep S. (2010). Current trends in biodegradable polyhydroxyalkanoates. J. Biosci. Bioeng..

[50] Curien G., Flori S., Villanova V., Magneschi L., Giustini C., Forti G., Matringe M., Petroutsos D., Kuntz M., Finazzi G. (2016). The water to water cycles in microalgae. Plant Cell Physiol..

[51] Zheng Y., Yu X., Li T., Xiong X., Chen S. (2014). Induction of D-xylose uptake and expression of NAD(P)H-linked xylose reductase and NADP + -linked xylitol dehydrogenase in the oleaginous microalga Chlorella sorokiniana. Biotechnol. Biofuels.

[52] Sudesh K., Abe H., Doi Y. (2000). Synthesis, structure and properties of polyhydroxyalkanoates: Biological polyesters. Prog. Polym. Sci..

[53] Hondo S., Takahashi M., Osanai T., Matsuda M., Hasunuma T., Tazuke A., Nakahira Y., Chohnan S., Hasegawa M., Asayama M. (2015). Genetic engineering and metabolite profiling for overproduction of polyhydroxybutyrate in cyanobacteria. J. Biosci. Bioeng..

[54] Saratale R.G., Cho S.-K., Saratale G.D., Kadam A.A., Ghodake G.S., Kumar M., Bharagava R.N., Kumar G., Kim D.S., Mulla S.I. (2021). A comprehensive overview and recent advances on polyhydroxyalkanoates (PHA) production using various organic waste streams. Bioresour. Technol..

[55] Bell I.R., Ives J.A., Wayne B.J. (2013). Nonlinear effects of nanoparticles: Biological variability from hormetic doses, small particle sizes, and dynamic adaptive interactions. Dose Response.

[56] Vranic S., Shimada Y., Ichihara S., Kimata M., Wu W., Tanaka T., Boland S., Tran L., Ichihara G. (2019). Toxicological evaluation of SiO_2_ nanoparticles by zebrafish embryo toxicity test. Int. J. Mol. Sci..

[57] McAdam B., Fournet M.B., McDonald P., Mojicevic M. (2020). Production of polyhydroxybutyrate (PHB) and factors impacting its chemical and mechanical characteristics. Polymers.

[58] Frone A.N., Nicolae C.A., Eremia M.C., Tofan V., Ghiurea M., Chiulan I., Radu E., Damian C.M., Panaitescu D.M. (2020). Low molecular weight and polymeric modifiers as toughening agents in poly(3-Hydroxybutyrate) films. Polymers.

[59] Magara G., Khan F.R., Pinti M., Syberg K., Inzirillo A., Elia A.C. (2019). Effects of combined exposures of fluoranthene and polyethylene or polyhydroxybutyrate microplastics on oxidative stress biomarkers in the blue mussel (*Mytilus edulis*). J. Toxicol. Environ. Health Part A.

[60] Zhao H., Cui Z., Sun X., Turng L.-S., Peng X. (2013). Morphology and properties of injection molded solid and microcellular polylactic acid/polyhydroxybutyrate-Valerate (PLA/PHBV) blends. Ind. Eng. Chem. Res..

[61] Abdelwahab M.A., Flynn A., Chiou B.-S., Imam S., Orts W., Chiellini E. (2012). Thermal, mechanical and morphological characterization of plasticized PLA–PHB blends. Polym. Degrad. Stab..

[62] Ansari N.F., Annuar M.S.M., Murphy B.P. (2016). A porous medium-chain-length poly(3-hydroxyalkanoates)/hydroxyapatite composite as scaffold for bone tissue engineering. Eng. Life Sci..

[63] Anbukarasu P., Sauvageau D., Elias A. (2015). Tuning the properties of polyhydroxybutyrate films using acetic acid via solvent casting. Sci. Rep..

[64] Sudesh K. (2012). Polyhydroxyalkanoates from Palm Oil: Biodegradable Plastics (SpringerBriefs in Microbiology).

[65] Janigová I., Lacík I., Chodák I. (2002). Thermal degradation of plasticized poly(3-hydroxybutyrate) investigated by DSC. Polym. Degrad. Stab..

[66] Thiré R.M.D.S.M., Arruda L.C., Barreto L.S. (2011). Morphology and thermal properties of poly(3-hydroxybutyrate-co-3-hydroxyvalerate)/attapulgite nanocomposites. Mater. Res..

[67] Corre Y.-M., Bruzaud S., Audic J.-L., Grohens Y. (2012). Morphology and functional properties of commercial polyhydroxyalkanoates: A comprehensive and comparative study. Polym. Test..

[68] Biradar G.G., Shivasharana C.T., Kaliwal B.B. (2018). Characterization of polyhydroxybutyrate (PHB) Produced by novel bacterium *Lysinibacillus sphaericus* BBKGBS6 isolated from soil. J. Polym. Environ..

[69] Costa S.S., Miranda A.L., Andrade B.B., Assis D.D.J., Souza C.O., de Morais M.G., Costa J.A.V., Druzian J.I. (2018). Influence of nitrogen on growth, biomass composition, production, and properties of polyhydroxyalkanoates (PHAs) by microalgae. Int. J. Biol. Macromol..

[70] Babruwad P.R., Prabhu S.U., Upadhyaya K.P., Hungund B.S. (2016). Production and characterization of thermostable polyhydroxybutyrate from *Bacillus cereus* PW3A. J. Biochem. Technol..

[71] Kovalcik A., Obruca S., Kalina M., Machovsky M., Enev V., Jakesova M., Sobkova M., Marova I. (2020). Enzymatic hydrolysis of poly(3-Hydroxybutyrate-*co*-3-Hydroxyvalerate) scaffolds. Materials.

[72] Assis D.D.J., Gomes G.V.P., Pascoal D.R.D.C., Pinho L.S., Chaves L.B.O., Druzian J.I. (2016). Simultaneous biosynthesis of polyhydroxyalkanoates and extracellular polymeric substance (EPS) from crude glycerol from biodiesel production by different bacterial strains. Appl. Biochem. Biotechnol..

[73] Lee S.N., Lee M.Y., Park W.H. (2002). Thermal stabilization of poly(3-hydroxybutyrate) by poly(glycidyl methacrylate). J. Appl. Polym. Sci..

[74] Koller M., Salerno A., Braunegg G. (2013). Polyhydroxyalkanoates: Basics, production and applications of microbial biopolyesters. Bio-Based Plastics: Materials and Applications.

[75] Anbukarasu P., Sauvageau D., Elias A.L. (2017). Enzymatic degradation of dimensionally constrained polyhydroxybutyrate films. Phys. Chem. Chem. Phys..

[76] Bhati R., Mallick N. (2015). Poly(3-hydroxybutyrate-co-3-hydroxyvalerate) copolymer production by the diazotrophic cyanobacterium *Nostoc muscorum* Agardh: Process optimization and polymer characterization. Algal Res..

[77] Napolitano S., Wübbenhorst M. (2006). Slowing down of the crystallization kinetics in ultrathin polymer films: A Size or an interface effect?. Macromolecules.

