# Supplementary material for: Characterization and Biotechnological Potential of Intracellular Polyhydroxybutyrate by Stigeoclonium sp. B23 Using Cassava Peel as Carbon Source
Source: Polymers (Basel). 2021 Feb 25;13(5):687. doi: 10.3390/polym13050687 (PMC7956423; doi:10.3390/polym13050687)
Supplement: Supplementary file 1 [file polymers-13-00687-s001.pdf]

polymers

Supplementary Materials

Characterization and Biotechnological Potential of Intracellular Polyhydroxybutyrate by *Stigeoclonium* sp. B23 Using Cassava Peel as Carbon Source

Murilo Moraes Mourão [1,*], Luciana Pereira Xavier [1], Ralph Urbatzka [2], Lucas Barbosa Figueiroa [3], Carlos Emmerson Ferreira da Costa [3], Carmen Gilda Barroso Tavares Dias [4], Maria Paula Cruz Schneider [5], Vitor Vasconcelos [2,6] and Agenor Valadares Santos [1,*]

[1] Laboratory of Biotechnology of Enzymes and Biotransformations, Institute of Biological Sciences, Federal University of Pará, 66075-110 Belém, Pará, Brazil; lpxavier@ufpa.br
[2] Interdisciplinary Center of Marine and Environmental Research—CIIMAR, University of Porto, 4450-208 Porto, Portugal; rurbatzka@ciimar.up.pt (R.U.); vmvascon@fc.up.pt (V.V.)
[3] Laboratory of Oils of the Amazon, Guamá Science and Technology Park, Federal University of Pará, 66075-750 Belém, Pará, Brazil; lucasfigueiroa57@gmail.com (L.B.F.); emmerson@ufpa.br (C.E.F.d.C.)
[4] Laboratory of Materials Processing, Institute of Technology, Federal University of Pará, 66075-110 Belém, Pará, Brazil; cgbtd@ufpa.br
[5] Genomics and Systems Biology Center, Federal University of Pará, 66075-110 Belém, Pará, Brazil; mariapaulacruzschneider@gmail.com
[6] Department of Biology, Faculty of Sciences, University of Porto, 4069-007 Porto, Portugal
* Correspondence: mouraomurilo@gmail.com (M.M.M.); avsantos@ufpa.br (A.V.S.)

Citation: Mourão, M.M.; Xavier, L.P..; Urbatzka, R.; Figueiroa, L.B.; Costa, C.E.F.d.; Dias, C.G.B.T.; Schneider, M.P.C.; Vasconcelos, V.; Santos, A.V. Characterization and Biotechnological Potential of Intracellular Polyhydroxybutyrate by *Stigeoclonium* sp. B23 Using Cassava Peel as Carbon Source. *Polymers* **2021**, *13*, 687. https://doi.org/10.3390/polym13050687

Academic Editor: Łukasz Klapiszewski

Received: 6 February 2021
Accepted: 17 February 2021
Published: 25 February 2021

Publisher's Note: MDPI stays neutral with regard to jurisdictional claims in published maps and institutional affiliations.

Copyright: © 2021 by the authors. Submitted for possible open access publication under the terms and conditions of the Creative Commons Attribution (CC BY) license (http://creativecommons.org/licenses/by/4.0/).

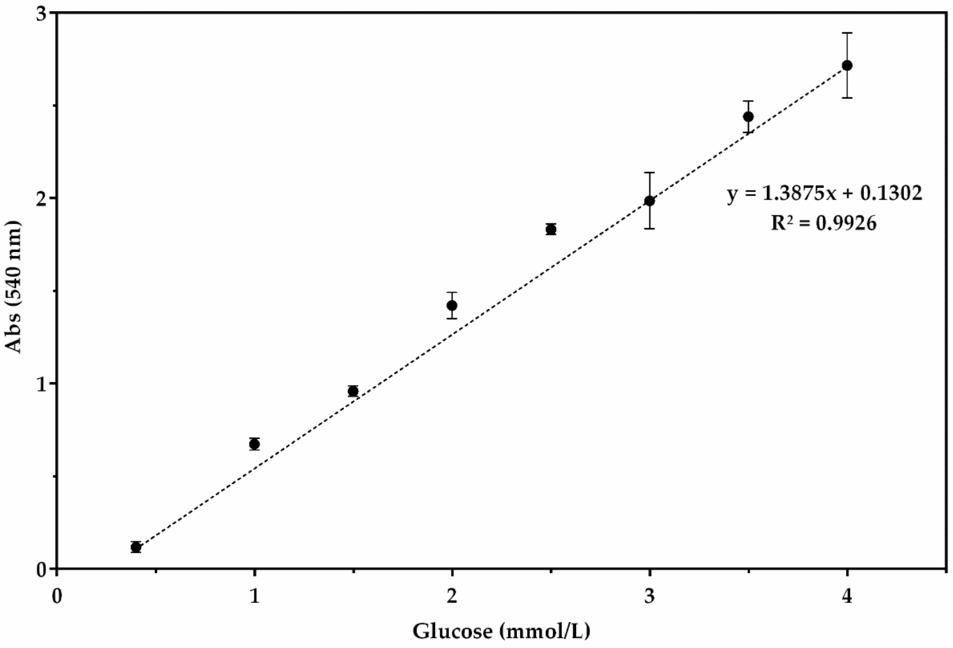

Figure S1. Standard curve of commercial glucose. UV absorbance 540 nm is plotted as a function of the amount of glucose in mmol/L. Data are the mean ± SD of three replicates.

Table S1. ANOVA Table of Calculated Parameter (BMP, PHB and P_{PHB}) and Modified Z8 Media of *Stigeoclonium* sp. B23 at the 95% Confidence Level.

	Degrees of Freedom	Sum of Squares	Mean of Squares	Fcalc	*p* Value
Interaction	4.0	144.2	36.04	58.83	$p < 0.0001$
Yield parameters	2.0	283.4	141.7	231.3	$p < 0.0001$
Modified Z8 media	2.0	58.07	29.03	47.39	$p < 0.0001$
Residual (error)	18.0	11.03	0.6126		
Total	26.0	496.7			

Table S2. Tukey's Post Hoc Multiple Comparisons of Calculated Parameters (BMP, PHB, and P_{PHB}) and Modified Z8 Media of *Stigeoclonium* sp. B23. The Mean Difference is Significant at the 0.05 level.

Treatment Comparisons	Mean Difference	95% Confidence Interval		*p* Value
		Lower Bound	Upper Bound	
**BMP (g/L)**				
Z8/100%$NaNO_3$ vs. Z8/25%$NaNO_3$	0.7273	0.8090	1.536	0.5039
Z8/100%$NaNO_3$ vs. Z8/2.5%$NaNO_3$	1.008	0.5282	1.536	0.2805
Z8/25%$NaNO_3$ vs. Z8/2.5%$NaNO_3$	0.2808	0.5282	0.8090	0.8996
**PHB (%)**				
Z8/100%$NaNO_3$ vs. Z8/25%$NaNO_3$	−11.24	12.17	0.9282	< 0.0001
Z8/100%$NaNO_3$ vs. Z8/2.5%$NaNO_3$	−7.977	8.905	0.9282	< 0.0001
Z8/25%$NaNO_3$ vs. Z8/2.5%$NaNO_3$	3.263	8.905	12.17	< 0.0001
**P_{PHB} (g/L)**				
Z8/100%$NaNO_3$ vs. Z8/25%$NaNO_3$	−0.08366	0.09792	0.01426	0.9906
Z8/100%$NaNO_3$ vs. Z8/2.5%$NaNO_3$	−0.03202	0.04628	0.01426	0.9986
Z8/25%$NaNO_3$ vs. Z8/2.5%$NaNO_3$	0.05164	0.04628	0.09792	0.9964
